# Inhibition of Pig Phosphoenolpyruvate Carboxykinase Isoenzymes by 3-Mercaptopicolinic Acid and Novel Inhibitors

**DOI:** 10.1371/journal.pone.0159002

**Published:** 2016-07-08

**Authors:** Jorge Hidalgo, Pedro Latorre, José Alberto Carrodeguas, Adrián Velázquez-Campoy, Javier Sancho, Pascual López-Buesa

**Affiliations:** 1 Departamento de Producción Animal y Ciencia de los Alimentos, Facultad de Veterinaria, Universidad de Zaragoza, Zaragoza, Spain; 2 Instituto de Biocomputación y Física de Sistemas Complejos (BIFI), BIFI-IQFR (CSIC) Joint Unit, Universidad de Zaragoza, Zaragoza, Spain; 3 Departamento de Bioquímica y Biología Molecular y Celular, Facultad de Ciencias, Universidad de Zaragoza, Zaragoza, Spain; 4 IIS Aragón, Zaragoza, Spain; 5 Fundación ARAID, Government of Aragón, Zaragoza, Spain; Weizmann Institute of Science, ISRAEL

## Abstract

There exist two isoforms of cytosolic phosphoenolpyruvate carboxykinase (PEPCK-C) in pig populations that differ in a single amino acid (Met139Leu). The isoenzymes have different kinetic properties, affecting more strongly the *K*_m_ and *V*_max_ of nucleotides. They are associated to different phenotypes modifying traits of considerable economic interest. In this work we use inhibitors of phosphoenolpyruvate carboxykinase activity to search for further differences between these isoenzymes. On the one hand we have used the well-known inhibitor 3-mercaptopicolinic acid. Its inhibition patterns were the same for both isoenzymes: a three-fold decrease of the K_i_ values for GTP in 139Met and 139Leu (273 and 873 μM, respectively). On the other hand, through screening of a chemical library we have found two novel compounds with inhibitory effects of a similar magnitude to that of 3-mercaptopicolinic acid but with less solubility and specificity. One of these novel compounds, (N'1-({5-[1-methyl-5-(trifluoromethyl)-1H-pyrazol-3-yl]-2-thienyl}methylidene)-2,4-dichlorobenzene-1-carbohydrazide), exhibited significantly different inhibitory effects on either isoenzyme: it enhanced threefold the apparent *K*_m_ value for GTP in 139Met, whereas in 139Leu, it reduced it from 99 to 69 μM. The finding of those significant differences in the binding of GTP reinforces the hypothesis that the Met139Leu substitution affects strongly the nucleotide binding site of PEPCK-C.

## Introduction

Mammalian cytosolic phosphoenolpyruvate carboxykinase (PEPCK-C, EC 4.1.1.32) catalyzes the GTP-dependent phosphorylation of OAA to yield PEP, GDP and CO_2_. The reverse reaction is possible, however it is unlikely to occur *in vivo* because the *K*_m_ for PEP is higher than its physiological concentrations [1–2]. PEPCK-C is situated in a crossroad of metabolic pathways; it is the main regulatory enzyme of gluconeogenesis and, in some tissues, of glyceroneogenesis. It also serves as regulator of the tricarboxylic acid cycle and is involved in serine biosynthesis [3]. Due to its gluconeogenic function, PEPCK-C activity favors pathological processes such as diabetes and cancer [4–7]. The involvement of PEPCK-C in glyceroneogenesis is convincingly illustrated by the supermouse generated by Hakimi et al. [8] in which a 100-fold overexpression of PEPCK-C in striated muscular tissue resulted in a 4-fold enhancement of intramuscular triglyceride content and a concomitant reduction of both visceral and abdominal fat.

We have recently found in pigs two isoforms of PEPCK-C differing in a single amino acid residue far away from the active center (Met139Leu) and exhibiting reduced and enhanced k_cat_ values in the gluconeogenic/glyceroneogenic reaction (OAA → PEP) and the reverse one (PEP → OAA), respectively [9]. Accordingly to the enzyme role in glyceroneogenesis, pigs carrying the 139Leu PEPCK-C (the one with lower k_cat_ values in the glyceroneogenic direction) had lower intramuscular fat content. Other negative phenotypic effects associated to the Met139Leu substitution in pigs were an increase in backfat thickness and lower meat quality due to enhanced postmortem exudation. The high frequency of 139Leu PEPCK-C alleles in modern pig breeds together with the set of negative effects associated to it makes the study of the pig isoenzymes of considerable economic relevance.

The main regulatory mechanism of PEPCK-C is transcriptional [10–11], although reversible acetylation of lysine residues has been shown to regulate also PEPCK-C stability *in vivo* [12]. In spite of its metabolic relevance, no known allosteric effector of PEPCK-C has been found to be physiologically relevant *in vivo*. Clearly, finding allosteric effectors of PEPCK-C could be interesting to modulate its activity in humans. In this respect, PEPCK-C inhibitors could be alternatives to ameliorate diabetes or to reduce high glucose levels needed by cancer cells [13].

The search for new ligands can focus on structural similarity of ligands with substrates or can be based on the known 3D structure of the enzyme, especially on its active site [14]. However, these strategies usually allow the finding of only inhibitors. Several such PEPCK-C inhibitors have been already described, most of which are structural analogues of substrates acting as competitive inhibitors that bind to the active site and few of them are non-competitive [15–17]. PEPCK-C inhibitors may help to investigate biochemical or functional differences between PEPCK-C isoenzymes. Alternatively, enzyme effectors can be identified by screening chemical libraries, a strategy that is being increasingly used to find molecules with a variety of biological effects [18–19].

In this work we pursue a double objective: to study more thoroughly the biochemical properties of pig PEPCK-C isoenzymes using a well-known inhibitor (3-mercaptopicolinic acid, 3-MP) and to identify new PEPCK-C effectors by screening a chemical library against the mentioned pig PEPCK-C isoenzymes.

## Materials and Methods

### 2.1. Enzymes and chemicals

Oxaloacetic acid (OAA), GTP, GDP, dithiothreitol (DTT), NADH, HEPES, 8-anilino-1-naphthalenesulfonic acid (ANS), dimethyl sulfoxide (DMSO), tris-(2-carboxyethyl) phosphine (TCEP), mineral oil and enzymes malate dehydrogenase (600–1000 units/mg), pyruvate kinase (600–1000 units/mL) and lactate dehydrogenase (900–1400 units/mL) were purchased from Sigma-Aldrich (USA). Phosphoenolpyruvate was obtained from Bachem (Switzerland). MnCl_2_, MgCl_2_ and KHCO_3_ were acquired from Panreac (Spain). 3-mercaptopicolinic acid (3-MP) was obtained from Sta. Cruz Biotechnology (USA). A chemical library of 10,000 compounds (HitFinder) and the subsequent positive compounds (N1-cyclohexyl-2-{1-[4-methyl-2-(2-thienyl)-1,3-thiazol-5-yl]ethylidene}hydrazine-1-carbothiamide, or compound 1, and N'1-({5-[1-methyl-5-(trifluoromethyl)-1H-pyrazol-3-yl]-2-thienyl}methylidene)-2,4-dichlorobenzene-1-carbohydrazide, or compound 2, were purchased from Maybridge (UK).

### 2.2. PEPCK-C purification

PEPCK-C 139Met and 139Leu were purified as described in Latorre et al. [9]. In brief, *Escherichia coli* BL21 (DE3) with the appropriate constructs were grown in LB + ampicillin medium and then protein expression was induced with IPTG. Clarified bacteria lysates were purified by affinity chromatography on cobalt column (GE Healthcare). Kinetic assays were performed with His-tagged PEPCK-C, which behaved almost identically to the non His-tagged enzyme [2]. For screening, CD spectroscopy and ITC measurements, the His-tag was removed by PreScission Protease (GE Healthcare) at 4°C overnight.

### 2.3. Screening of chemical library

PEPCK-C effectors were identified from a chemical library using a screening procedure based on a thermal-shift assay [20–21]. Potential protein effector compounds were dispensed into 96-well microplates. Each well contained 4 μM PEPCK-C in a 20 mM HEPES buffer pH 7.4, plus 1 mM TCEP, 100 μM ANS, 10% DMSO and either 1 or 5 chemical library compounds, each at a 25 μM concentration. Previous experiments showed that PEPCK-C tolerates up to 12% DMSO (data not shown). 50 μL of mineral oil were added to each well to prevent evaporation. In a first screening round, 5 different compounds were present in each well. The binding of effectors to PEPCK-C was assessed by monitoring the thermal denaturation of the enzyme and identifying compounds inducing a stabilizing effect (increase in the unfolding temperature, T_m_, compared to the free enzyme) in a FluoDia T70 High Temperature Fluorescence Microplate Reader (Photon Technology International, UK). To obtain T_m_ values, unfolding curves were fitted to Boltzmann function (Eq 1) using OriginPro 9.1 software.

$$y\left(T\right)\;=\;\frac{A_{1}-A_{2}}{1+exp\left(\frac{T-T_{m}}{S}\right)}+A_{2}$$ (1)

where A_1_ and A_2_ are the asymptotic values of the signal in the pre- and post-transition region of the unfolding curve, respectively, and S is a parameter associated with the slope of the unfolding curve at the T_m_ and related to the unfolding enthalpy of the enzyme. A given well was considered as a positive hit if its associated T_m_ value was 8°C higher than the T_m_ found in control wells lacking added library compounds. In a second round, the compounds present in the positive wells were individually analyzed to identify the binders.

### 2.4. Enzymatic assays

PEPCK-C activity was measured in an UNICAM 500 spectrometer (UNICAM Analytical Systems, UK) with coupled spectrophotometric assays using pyruvate kinase and lactate dehydrogenase as auxiliary enzymes in PEP synthesis reaction and malate dehydrogenase in the reverse reaction.

Assays to calculate IC_50_ values were performed in the direction of OAA synthesis in 1 mL of a 100 mM HEPES buffer pH 7.4, containing 10 mM DTT, 0.2 mM MnCl_2_, 2 mM MgCl_2_, 2 mM GDP, 0.2 mM NADH, 2 mM PEP, 100 mM KHCO_3_ and 4 units of malate dehydrogenase. Variable inhibitor concentrations of 0–200 μM were also used. Assays were initiated by PEPCK-C addition. Rates were calculated by measuring the decreasing slope of absorbance at 340 nm after subtracting the rate of spontaneous NADH oxidation.

3-MP inhibitions assays in OAA synthesis direction were carried out as described before with the exception of the variable substrate (50–600 μM for PEP and 10–100 μM for GDP) and 3-MP concentration (10–100 μM). Assays in the direction of PEP synthesis were performed in 1 mL of a 100 mM HEPES buffer pH 7.4, containing 10 mM DTT, 0.2 mM MnCl_2_, 2 mM MgCl_2_, 1 mM GTP or 400 μM OAA, 1 mM ADP, 0.2 mM NADH, PEPCK-C and 5 units of each pyruvate kinase and lactate dehydrogenase. Reactions were initiated by addition of OAA. Variable substrates were 5–50 μM OAA or 25–500 μM GTP and 3-MP concentrations were between 10–400 μM. Rates were calculated by measuring the decreasing slope at 340 nm of absorbance after subtracting the rate of spontaneous NADH oxidation in the case of OAA synthesis or the rate of the blank which contained all the components of the mix but PEPCK-C in the case of PEP synthesis.

To investigate the possible inhibition of auxiliary enzymes by compound 1 and 2 the following assay was performed in 1 mL of a 100 mM HEPES buffer pH 7.4, containing 10 mM DTT, 0.2 mM MnCl_2_, 2 mM MgCl_2_, 2 mM GDP, 0.2 mM NADH, 2 mM PEP, 1 mM ADP, 100 mM KHCO_3_ and 25–200 μM compound 1 or 2. Reactions were initiated by addition of 0.5 units of each pyruvate kinase and lactate dehydrogenase.

Compound 1 and 2 inhibitions assays were performed in PEP synthesis direction as described in 3-MP inhibition assays. Variable substrates were 10–100 μM OAA or 25–500 μM GTP, inhibitors concentrations were around IC_50_ value (100 μM compound 1 or 50 μM compound 2) and 20 units of each pyruvate kinase and lactate dehydrogenase were also added. The units of pyruvate kinase and lactate dehydrogenase were increased four times in these assays due to slight interaction of compounds 1 and 2 with these enzymes.

### 2.5. Determination of kinetic parameters

IC_50_ value represents the inhibitor concentration required to reduce activity by 50%. Concentration-dependent inhibition data were fitted to a linear or a quadratic function using GraphPad Prism 3.03 software. 3-MP inhibition assays were fitted in double reciprocal plots. To describe the mixed non-competitive inhibition, the double reciprocal equation can be written as:

$$\frac{1}{v}\;=\;\frac{K_{m}}{V_{max}}\;\left(1+\frac{\left[I\right]}{K_{i}}\right)\;\frac{1}{\left[S\right]}+\frac{1}{V_{max}}\;\left(1+\frac{\left[I\right]}{\alpha K_{i}}\right)$$ (2)

Secondary plots to calculate K_i_ and αK_i_ values can be constructed from:

$$Slope\;=\;\frac{K_{m}}{V_{max}}+\frac{K_{m}\left[I\right]}{V_{max}K_{i}}$$ (3)

$$Y-intercept\;=\;\frac{1}{V_{max}}+\frac{1}{\alpha K_{i}V_{max}}\left[I\right]$$ (4)

K_i_ and αK_i_ are the dissociation constants of the enzyme-inhibitor complex and the enzyme-inhibitor-substrate complex, respectively.

K_i_ values with respect to GTP and GDP were calculated by Dixon plots with all substrates at saturating concentration because 3-MP produced an uncompetitive inhibition. The equation for the Dixon plot is:

$$\frac{1}{v}\;=\;\frac{K_{m}}{V_{max}\left[S\right]}+\frac{1}{V_{max}}+\frac{K_{m}}{V_{max}\left[S\right]K_{i}}$$ (5)

Data from compound 1 and 2 inhibition assays were represented in double reciprocal plots to calculate *V*_max_ and *K*_m_ values using GraphPad Prism 3.03 software.

### 2.6. Circular dichroism (CD)

PEPCK-C was diluted to 20 μM in a 20 mM HEPES pH 7.4 buffer, containing 1 mM TCEP. 50 μM 3-MP, compound 1 or compound 2 were added to the PEPCK-C solution. Readings were performed at 25°C in a 0.1 cm cuvette from 320 to 250 nm in a Chirascan CD-spectrophotometer (Applied Photophysics, UK). CD spectra were calculated after subtracting the blank which contained all the components except PEPCK-C.

### 2.7. Isothermal titration calorimetry (ITC)

Isothermal titration calorimetry (ITC) experiments were performed using an Auto-iTC200 microcalorimeter (MicroCal, Malvern, UK) at 25°C. The reference cell was filled with distilled water. The sample cell was loaded with 10 μM PEPCK-C 139M or 139L in a 20 mM HEPES pH 7.4 buffer, containing 150 mM NaCl, 1 mM TCEP and 6.7% DMSO. 2 mM GTP was also added to the enzyme solution in the corresponding experiments. The syringe was loaded with 400 μM 3-MP or 100 μM compound 2 in a 20 mM HEPES pH 7.4 buffer, containing 150 mM NaCl, 1 mM TCEP and 6.7% DMSO. In all the assays, a total of 19 injections of 2 μL were added sequentially to the sample cell with a 150 s spacing to ensure that the thermal power signal returned to the baseline prior to the next injection. The binding isotherms (normalized heat effect as a function of the molar ratio) were analyzed with Origin 7 (OriginLab) using a single class of binding sites model. Calorimetric assays were perform in duplicates at least.

### 2.8. Statistical analysis

Comparisons between 139Met and 139Leu K_i_ and between 139Met and 139Leu αK_i_ in 3-MP kinetics were performed using two-sample *t-*tests. p<0.05 was considered statistically significant. Compound 1 and 2 kinetic experiments were also analyzed using two-sample *t*-tests comparing 139Met and 139Leu *K*_m_ at the same inhibition range (0 or 50 μM) and 139Met and 139Leu *V*_max_ at the same inhibition range (0 or 50 μM). p<0.05 was considered statistically significant.

## Results

### 3.1. Inhibition with 3-MP

To investigate the biochemical properties of porcine PEPCK-C isoenzymes we used a well-known PEPCK-C inhibitor, 3-MP. We first determined IC_50_ values of wild type PEPCK-C at saturating substrate concentration in the direction of OAA synthesis. An IC_50_ value of 65 ± 6 μM was found (S1 Fig). This value is useful to select the inhibitor concentration range in further kinetic experiments.

We then studied the inhibition patterns by 3-MP of the two PEPCK-C isoenzymes.

**Fig 1:**
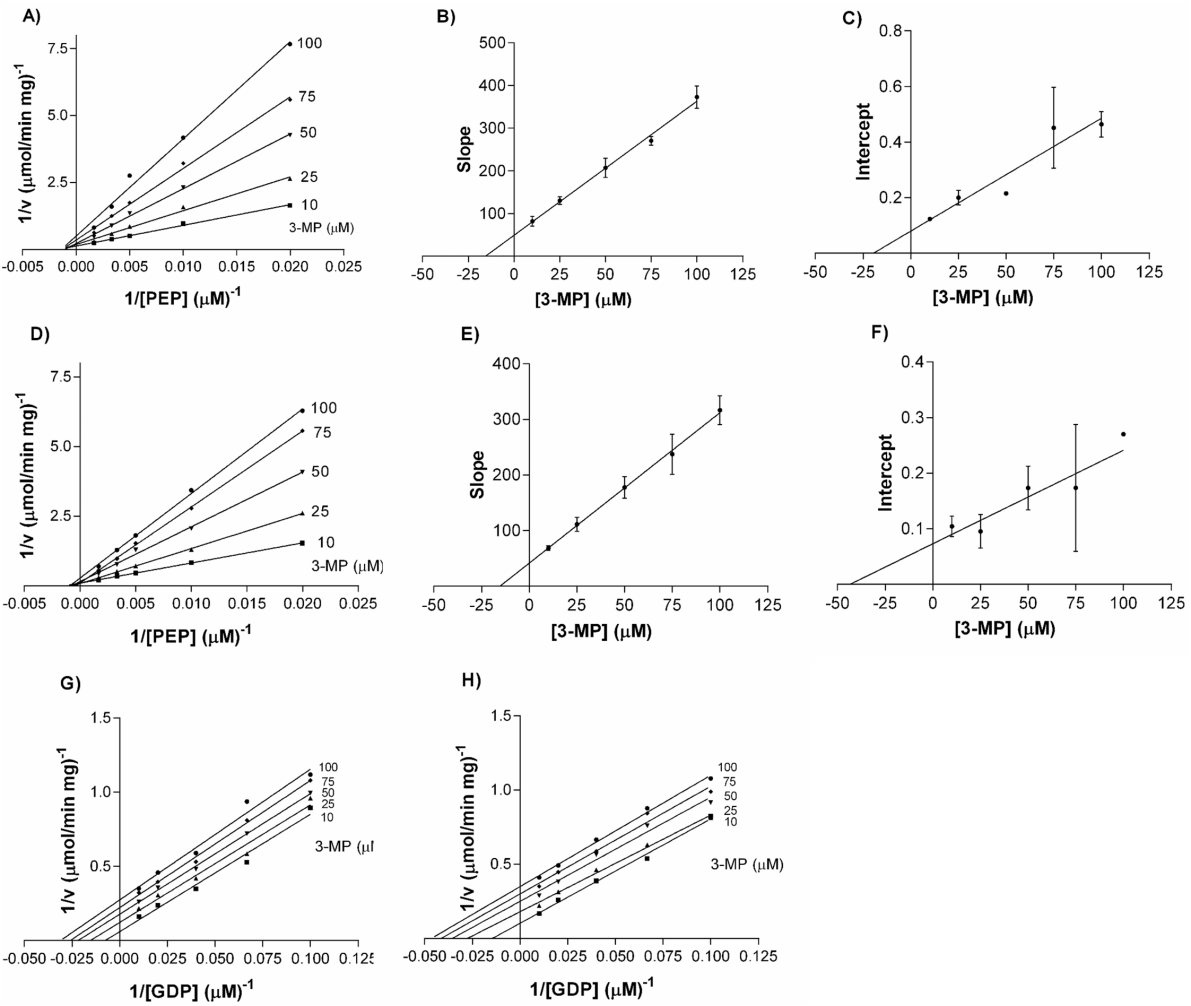
Double reciprocals and secondary plots of 3-MP adding PEP and GDP as variable substrates. (A-C) Double reciprocal, slopes and intercepts of 139Met varying PEP. (D-F) Double reciprocal, slopes and intercepts of 139Leu varying PEP. (G) Double reciprocal of 139Met varying GDP. (H) Double reciprocal of 139Leu varying GDP. 139Met and 139Leu were assayed as described in Materials and Methods with increasing concentration of substrates (50–600 μM for PEP, 10–100 μM for GDP) and 3-MP concentration between 10–100 μM.

The patterns were different for the two substrates of the reverse reaction (OAA synthesis); whereas 3-MP behaved as a mixed non-competitive inhibitor when PEP was used as variable substrate, it behaved as an uncompetitive inhibitor when GDP was used as a variable substrate (Fig 1 and S2 Fig).

**Fig 2:**
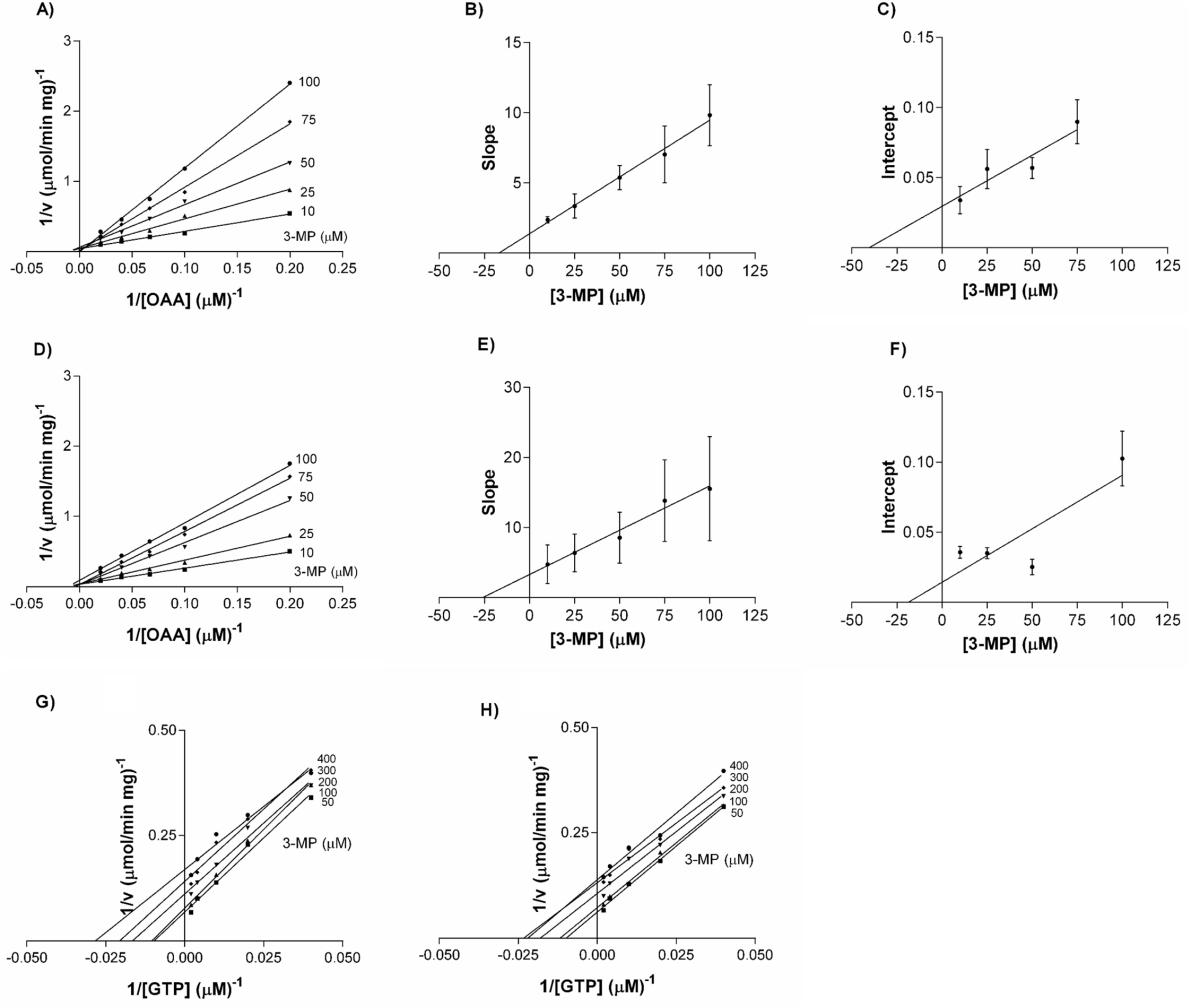
Double reciprocals and secondary plots of 3-MP adding OAA and GTP as variable substrates. (A-C) Double reciprocal, slopes and intercepts of 139Met varying OAA. (D-F) Double reciprocal, slopes and intercepts of 139Leu varying OAA. (G) Double reciprocal of 139Met varying GTP. (H) Double reciprocal of 139Leu varying GTP.139Met and 139Leu were assayed as described in Materials and Methods with increasing concentration of substrates (5–50 μM for OAA, 25–500 μM for GTP) and 3-MP concentration between 10–100 μM for OAA and between 50–400 μM for GTP.

When the effect of 3-MP was studied on the glyceroneogenic reaction, the inhibition patterns were exactly the same as the observed in the reverse reaction; 3-MP behaved as an uncompetitive inhibitor when using a nucleotide (GTP in this case) as a variable substrate and as a mixed non-competitive inhibitor when using OAA as a variable substrate (Fig 2 and S3 Fig).

We found no significant differences between 139Met and 139Leu in the reverse reaction. PEP K_i_ values were 16 ± 2 μM and 19 ± 4 μM and αK_i_ values were 20 ± 4 μM and 31 ± 5 μM for 139Met and 139Leu, respectively. K_i_ values with respect to GDP were 45 μM for both isoenzymes.

K_i_ values for glyceroneogenic substrates showed significant differences: they were 3 and 1.6 fold lower for 139Met when using GTP and OAA as variable substrates, respectively. However, αK_i_ value with respect to OAA showed no significant differences. All the K_i_ and αK_i_ values are presented in Table 1.

**Table 1:** K_i_ and αK_i_ values and inhibition patterns of PEPCK-C isoenzymes in presence of 3-MP.

	PEP			GDP		OAA			GTP	
	K_i_ (μM)	αK_i_ (μM)	Pattern of inhibition	K_i_ (μM)	Pattern of inhibition	K_i_ (μM)	αK_i_ (μM)	Pattern of inhibition	K_i_ (μM)	Pattern of inhibition
139Met	16 ± 2	20 ± 4	Mixed NC	45 ± 3	Uncompetitive	18 ± 4	49 ± 18	Mixed NC	273 ± 68	Uncompetitive
139Leu	19 ± 4 *ns*	31 ± 5 *ns*	Mixed NC	45 ± 0.8 *ns*	Uncompetitive	29 ± 1 *	21 ± 13 *ns*	Mixed NC	873 ± 144 **	Uncompetitive

Values given are the mean ± SD. Differences were calculated using two-sample *t*-test; ns: no significant,

* p< 0.05,

** p< 0.01.

NC: Non-Competitive

### 3.2. Screening of chemical library

**Fig 3:**
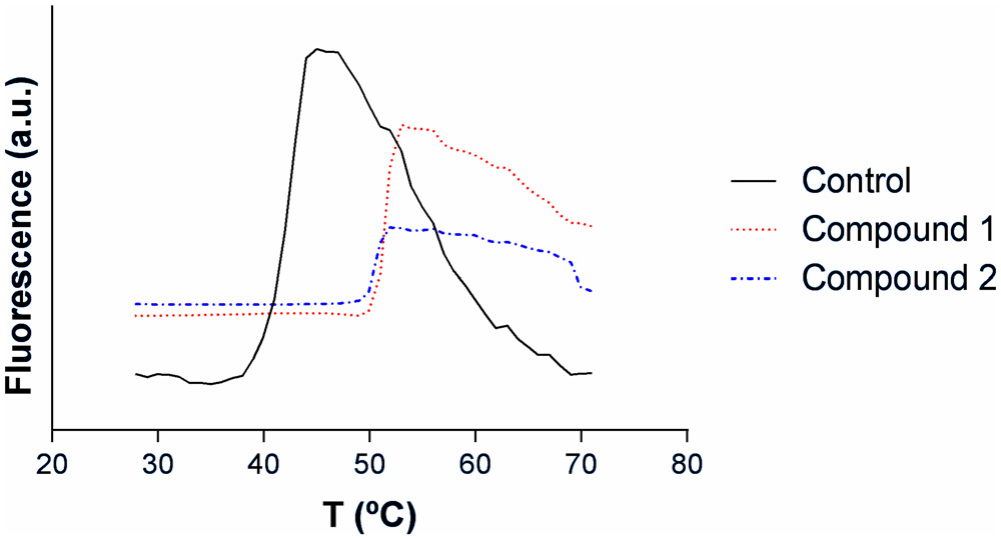
Thermal unfolding of PEPCK-C in presence and absence of compound 1 or 2.

To search for new PEPCK-C effectors we used a 10,000-compound chemical library from Maybridge and the 139Met enzyme. Initial screening was performed using 5 compounds per well. Afterwards, compounds in positive wells were analyzed individually. A change in unfolding temperature, measured by fluorescence, was used as indicator for a positive hit (see Fig 3). In the first round 7 hits were identified with T_m_ values of 55.5 ± 0.1, 56.4 ± 0.3, 48.1 ± 0.1, 53.9 ± 0.2, 56.9 ± 0.2, 53.0 ± 0.3 and 52.6 ± 0.1°C, well above the unfolding temperature determined in control wells (41.8 ± 0.1°C).

The 35 compounds contained in the 7 positive wells of the first screening were then analyzed individually. In this screening we found only two hits with T_m_ values of 50.9 ± 0.1 and 50.0 ± 0.1, which is about 8°C higher than controls. The absence of positive hits in the remainder wells can be attributed to a previous accumulative stabilizing effect of the compounds in the initial positive wells or just be false positives.

**Fig 4:**
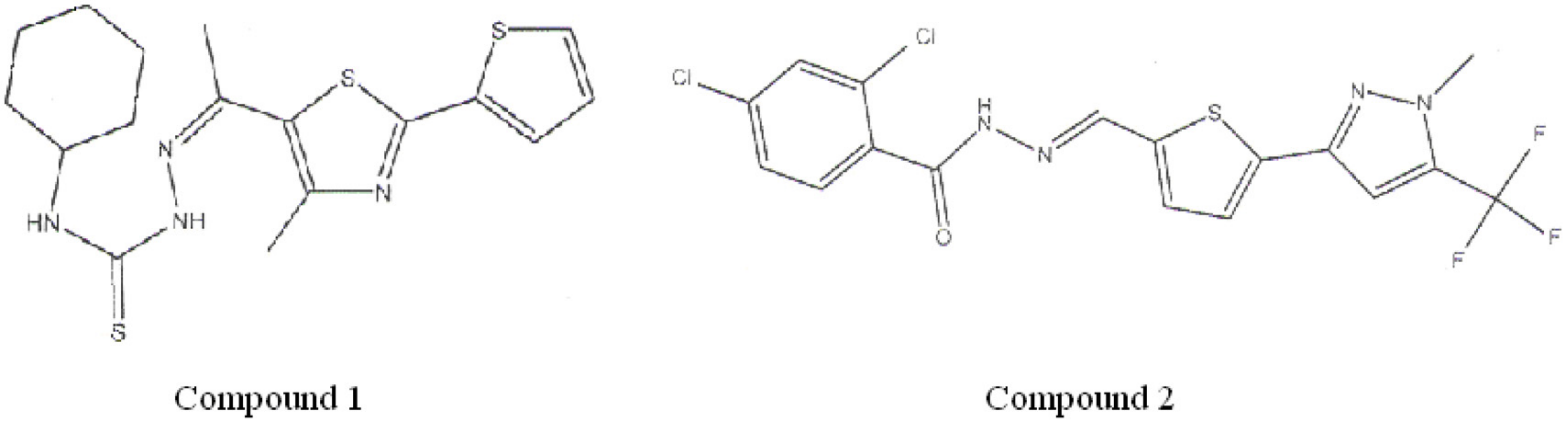
Structural formula of compounds 1 and 2.

The identified compounds were N1-cyclohexyl-2-{1-[4-methyl-2-(2-thienyl)-1,3-thiazol-5-yl]ethylidene}hydrazine-1-carbothiamide, or compound 1, and N'1-({5-[1-methyl-5-(trifluoromethyl)-1H-pyrazol-3-yl]-2-thienyl}methylidene)-2,4-dichlorobenzene-1-carbohydrazide, or compound 2 (Fig 4). Compounds 1 and 2 were selected for subsequent kinetic analysis.

### 3.3. Inhibition with compound 1

We first calculated IC_50_ values at saturating substrate concentrations assaying the activity in the direction of OAA synthesis. Compound 1 showed an IC_50_ value of 134 ± 4 μM (S4 Fig). This value was higher than that obtained for 3-MP (65 ± 6 μM. Concentrations above 250 μM resulted in precipitation of compound 1 due to its poor solubility. Compound 1 additionally showed a slight inhibition of the auxiliary enzymes pyruvate kinase and lactate dehydrogenase (data not shown), which was solved by increasing four times the concentration of the auxiliary enzymes. Due to the mentioned low solubility problems we decided to perform for compound 1 a more simple analysis of inhibition than the one performed with 3-MP. We calculated only the change in *V*_max_ and *K*_m_ values, instead of K_i_ values, using an inhibitor concentration around its IC_50_.

**Fig 5:**
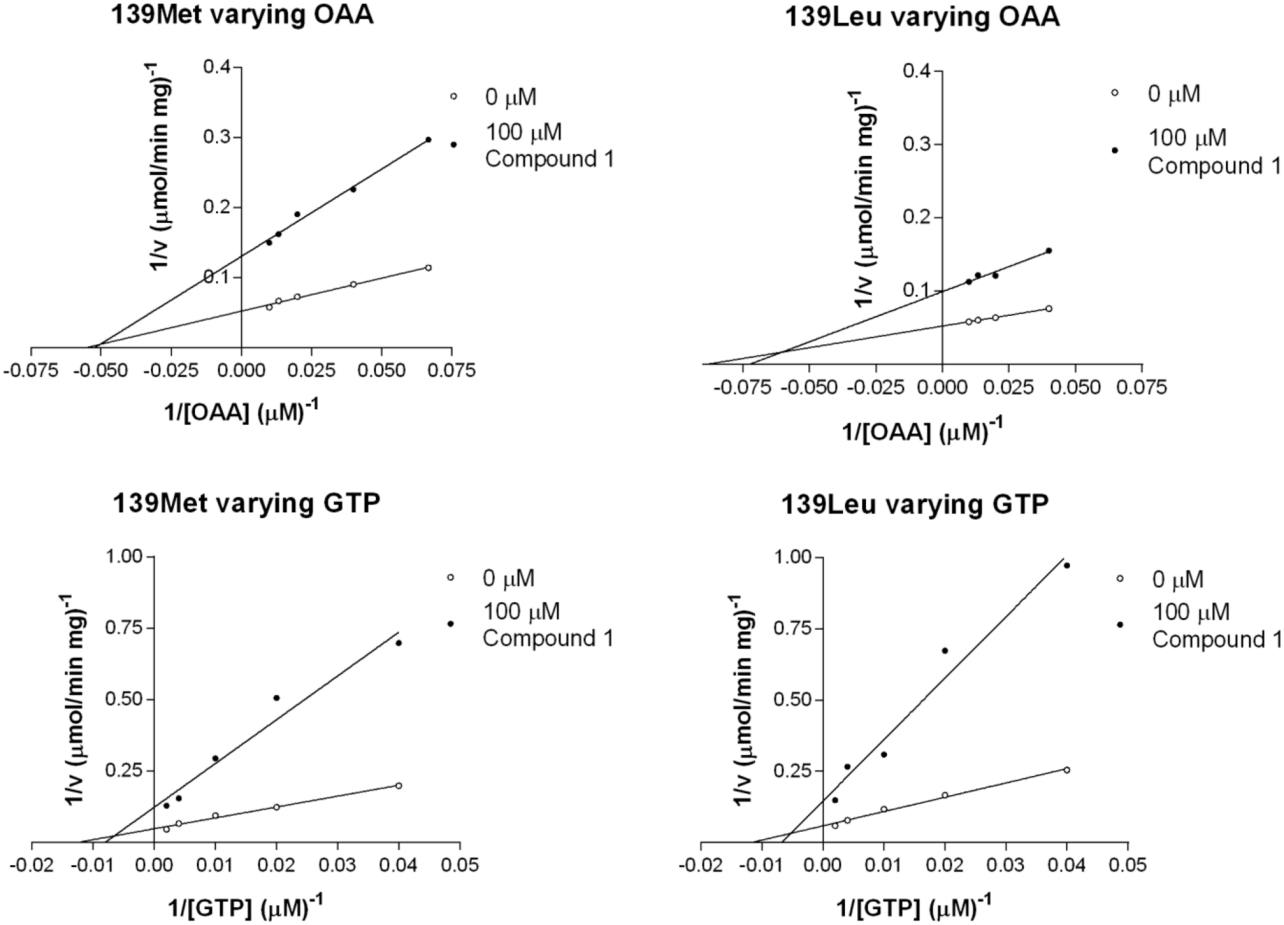
Double reciprocal plots of compound 1 inhibition varying glyceroneogenic substrates.

When using OAA as variable substrate we found a drop in *V*_max_ value (from 21.3 to 6.7 μmol min^-1^ mg^-1^ in 139Met and from 21.8 to 9.4 μmol min^-1^ mg^-1^ in 139Leu) and no change in *K*_m_ (Fig 5). There were no significant differences in *V*_max_ or *K*_m_ between 139Met and 139Leu (Table 2).

**Table 2:** *V*_max_ and *K*_m_ values and inhibition patterns of PEPCK-C isoenzymes in the presence of glyceroneogenic substrates and compound 1.

	[Compound 1] (μM)	OAA		GTP	
		*V*_max_ (μmol min^-1^ mg^-1^)	*K*_m_ (μM)	*V*_max_ (μmol min^-1^ mg^-1^)	*K*_m_ (μM)
139Met	0 μM	21.3 ± 3.7	22 ± 14	20.0 ± 1.9	96 ± 15
139Met	50 μM	6.7 ± 1.4	13 ± 5	7.8 ± 0.5	118 ± 14
139Leu	0 μM	21.8 ± 1.8 *ns*	14 ± 4 *ns*	22.5 ± 4.2 *ns*	99 ± 14 *ns*
139Leu	50 μM	9.4 ± 1.3 *ns*	11 ± 3 *ns*	7.2 ± 0.4 *ns*	155 ± 30 *ns*

Values given are the mean of three independent experiments ± SD. Differences in *V*_max_ and *K*_m_ between 139Met and 139Leu were calculated using two-sample *t*-test; ns: not significant.

Nevertheless, when GTP was used as variable substrate *V*_max_ values were reduced from 20.0 to 7.8 μmol min^-1^ mg^-1^ and from 22.5 to 7.2 μmol min^-1^ mg^-1^ for 139Met and 139Leu, respectively. *K*_m_ values raised from 96 and 99 to 118 and 155 μM in 139Met and 139Leu, respectively. There were also no significant differences between PEPCK-C isoenzymes (Table 2).

### 3.4. Inhibition with compound 2

IC_50_ value for compound 2 was 30 ± 2 μM (S4 Fig). This value was lower than those of 3-MP (65 ± 6 μM) or compound 1 (134 ± 4 μM). Compound 2 also presented poor solubility and a slight interaction with auxiliary enzymes, what made necessary a four-fold increase in the amount of auxiliary enzymes added to the assays.

**Fig 6:**
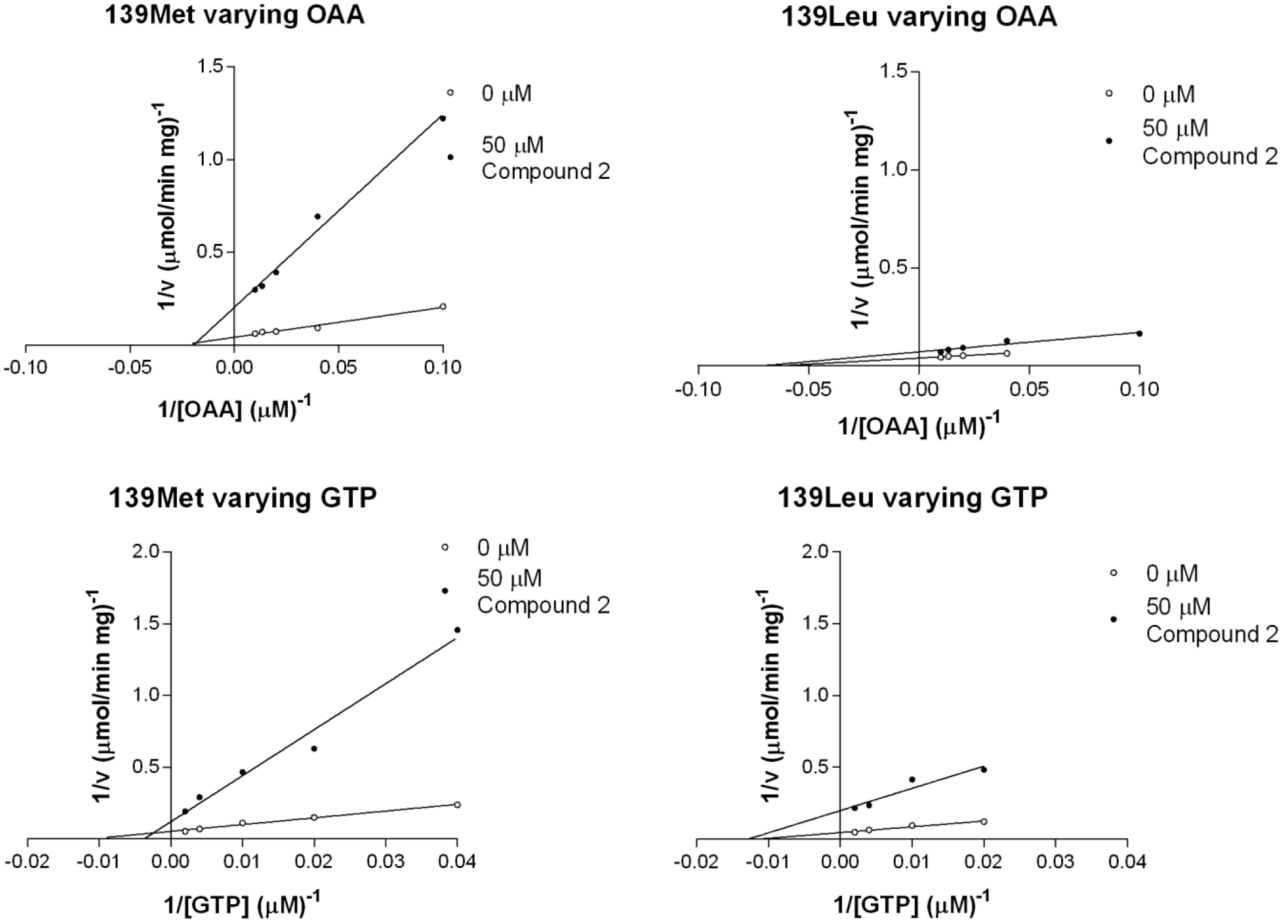
Double reciprocal plots of compound 2 inhibition varying glyceroneogenic substrates.

*V*_max_ values for OAA in the presence of the inhibitor were reduced from 21.3 to 4.6 μmol min^-1^ mg^-1^ in the case of 139Met and from 21.8 to 12.7 μmol min^-1^ mg^-1^ in the case of 139Leu. This change was significantly different between 139Met and 139Leu, the former isoform being more sensitive to inhibition by compound 2. *K*_m_ values remained constant (Fig 6).

When using GTP as a variable substrate, we found significant differences between isoenzymes: whereas 139Met *K*_m_ value was raised in the presence of the inhibitor over 2.5 fold, 139Leu *K*_m_ value was reduced by about 30% (see Table 3). No significant change was observed in the effect of compound 2 on *V*_max_ values of 139Met and 139Leu.

**Table 3:** *V*_max_ and *K*_m_ values and inhibition patterns of PEPCK-C isoenzymes in the presence of glyceroneogenic substrates and compound 2.

	[Compound 2] (μM)	OAA		GTP	
		*V*_max_ (μmol min^-1^ mg^-1^)	*K*_m_ (μM)	*V*_max_ (μmol min^-1^ mg^-1^)	*K*_m_ (μM)
139Met	0 μM	21.3 ± 3.7	22 ± 14	20.0 ± 1.9	96 ± 15
139Met	50 μM	4.6 ± 1.4	32 ± 19	5.9 ± 2.3	276 ± 37
139Leu	0 μM	21.8 ± 1.8 *ns*	14 ± 4 *ns*	22.5 ± 4.2 *ns*	99 ± 14 *ns*
139Leu	50 μM	12.7 ± 1.1 **	12 ± 4 *ns*	6.1 ± 1.0 *ns*	69 ± 10 ***

Values given are the mean of three independent experiments ± SD. Differences in *V*_max_ and *K*_m_ between 139Met and 139Leu were calculated using two-sample *t*-test;

ns: not significant,

** p< 0.01

*** p< 0.001

### 3.5. CD spectra

**Fig 7:**
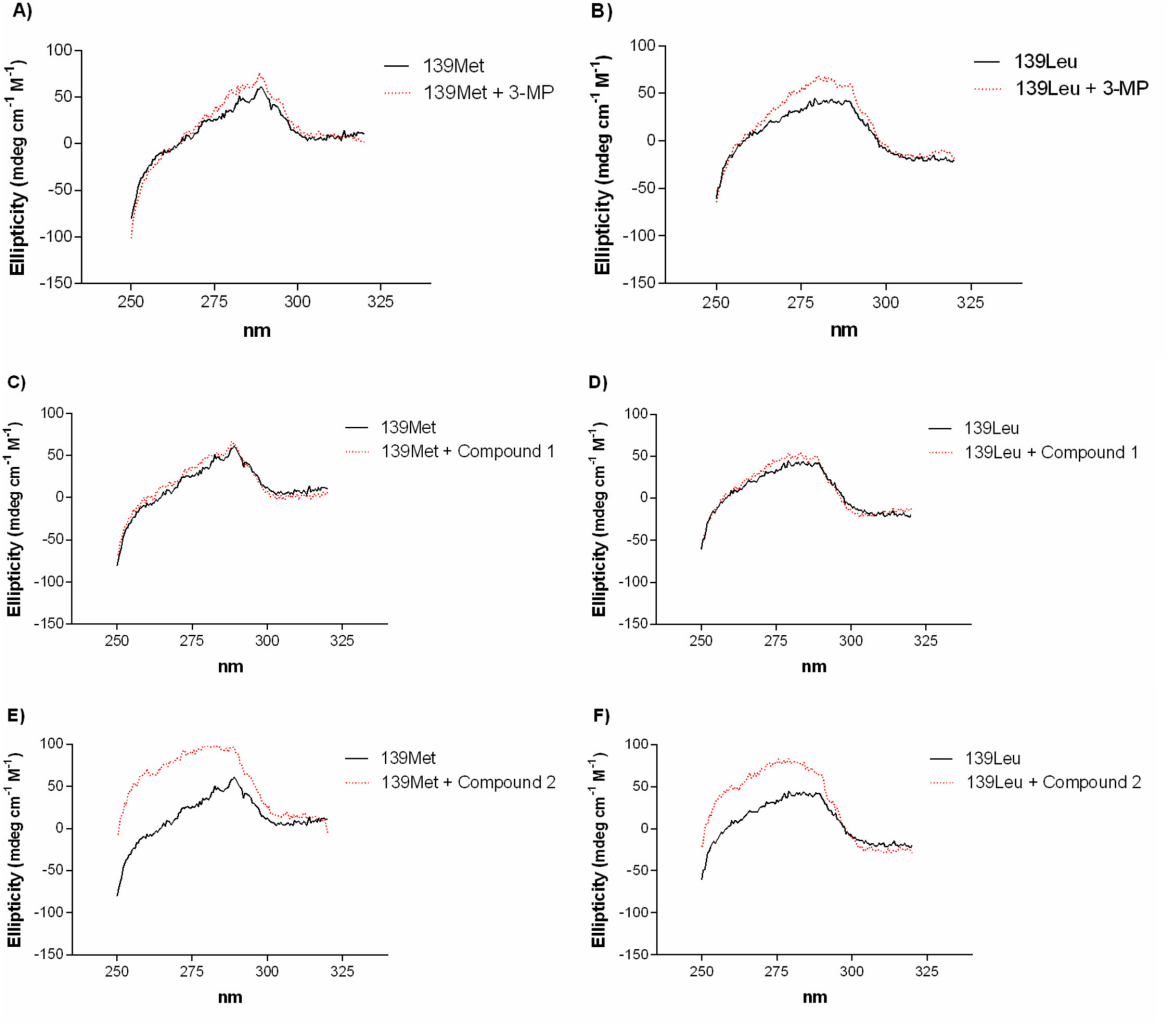
Near-UV CD spectra of PEPCK-C isoenzymes. 50 μM 3-MP (A-B), compound 1 (C-D) or compound 2 (E-F) were added to 20 μM PEPCK-C in a 20 mM HEPES pH 7.4 buffer and 1 mM TCEP.

The near UV-CD spectra of a protein arises from contributions of aromatic residues in asymmetric environments, which is characteristic of native protein conformations [22], and it is very sensitive to small changes such as the binding of compounds near those aromatic residues. Therefore modifications of the near-UV CD spectrum of a protein in presence of a given compounds reveals binding. However, the reverse needs not be necessarily true, because binding can occur far away from contributing aromatic residues. To find further proof that 3-MP, compound 1 and compound 2 bind to PEPCK-C, the near-UV CD spectra of the enzymes in presence of those compounds were recorded. As can be seen in Fig 7, 3-MP and more markedly compound 2 modify the CD spectra of both isoenzymes. However, there were no clear differences in the enzyme CD spectra when 50 μM (Fig 7C and 7D) or 150 μM (data not shown) of compound 1 was added to a PEPCK-C solution. This may be indicative of, although it does not prove it, a weaker binding of compound 1 to PEPCK-C, compared to 3-MP and compound 2, as additionally suggested by its weaker inhibitory effect.

### 3.6. Isothermal titration calorimetry (ITC)

**Fig 8:**
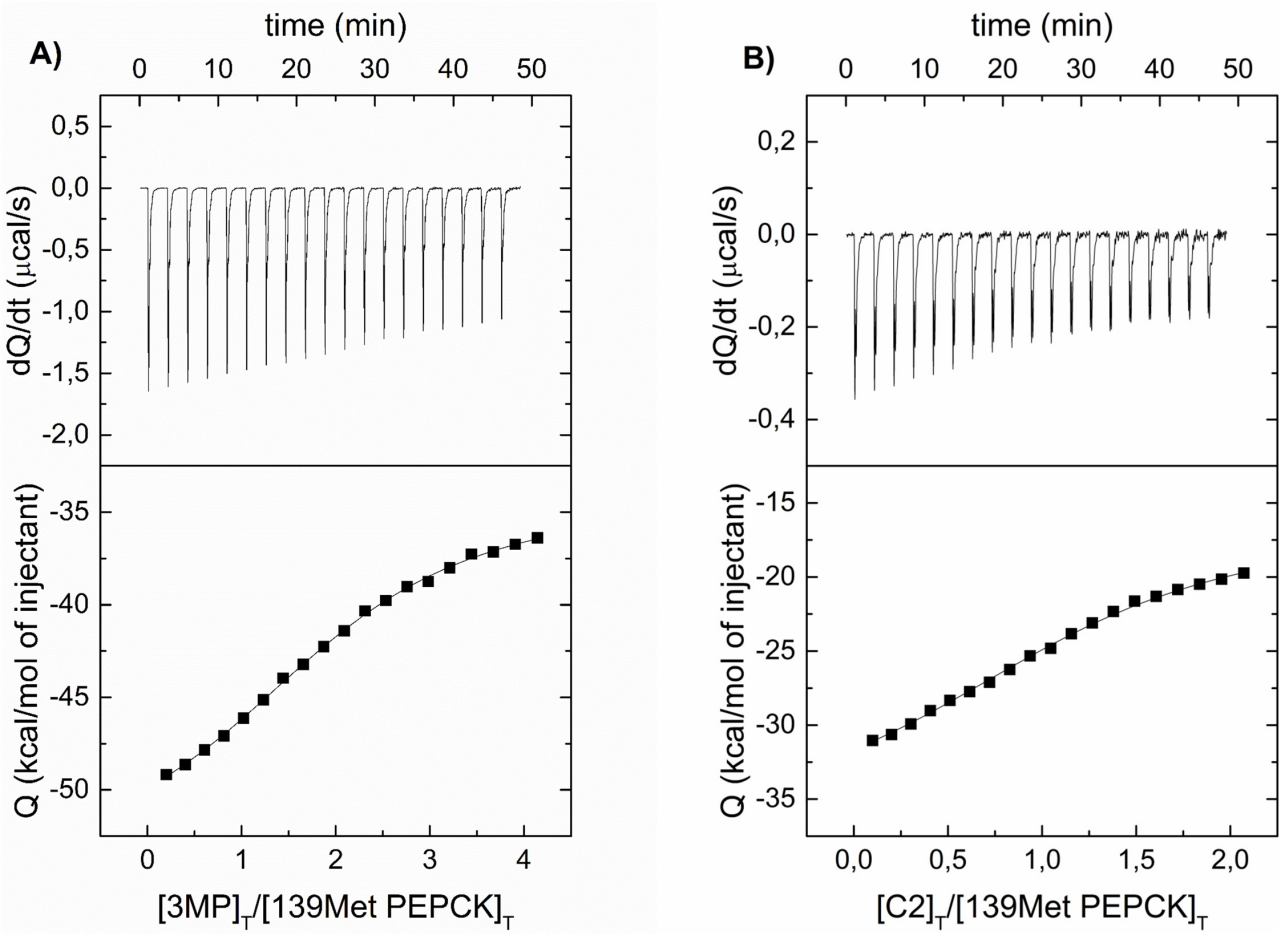
ITC measurements of PEPCK-C isoenzymes with 3-MP or compound 2. The upper part of each panel shows the thermogram after baseline correction, and the lower part shows the binding isotherm with the fit to a single class of binding sites model. (A) 139Met with 3-MP. (B) 139Met with compound 2.

In order to prove that Met139Leu substitution and the inhibitor binding affects the nucleotide binding site, we performed ITC experiments in the presence or the absence of nucleotide. The results of these experiments are shown in Fig 8 and the derived thermodynamic parameters are summarized in Table 4. We have compared 139Met and 139Leu, 3-MP and compound 2, both in the presence and the absence of GTP. We have chosen GTP because the kinetic differences between isoenzymes were larger with GTP than with GDP.

**Table 4:** ITC derived thermodynamic parameters.

			K_d_ (μM)	ΔH (kcal/mol)	n
139Met	3-MP	-GTP	7.2	-20.4	2.0
	3-MP	+GTP	19	-31.8	2.1
	C2	-GTP	4.4	-20.5	1.2
	C2	+GTP	19	-26.6	1.1
139Leu	3-MP	-GTP	12	-32.6	2.1
	3-MP	+GTP	39	-58.2	2.1
	C2	-GTP	9.8	-16.7	1.2
	C2	+GTP	14	-13.9	1.2

Relative error in K_d_ is 20%, absolute error in ΔH is 0.6 kcal/mol, and absolute error in n is 0.1.

The dissociation constant (K_d_) describes the affinity between a protein and a ligand. In all experiments, the Met139Leu substitution reduces the affinity of the inhibitors (Table 4). In the case of 3-MP, K_d_ values are 7.2 μM and 12 μM for 139Met and 139Leu, respectively, whereas in the case of compound 2 K_d_ values are 4.4 μM for 139Met and 9.8 for 139Leu. The presence of GTP further reduces the affinity of the 3-MP (19 μM for 139Met and 39 μM for 139Leu). Nevertheless, the presence of GTP raises the affinity of the compound 2 (19 μM for 139Met and 14 μM for 139Leu), although K_d_ values are different in presence and absence of GTP.

Enthalpy (ΔH) follows the same pattern of differences showed in K_d_ values. The negative enthalpy produced in all experiments showed that the binding of 3-MP and compound 2 are enthalpically driven.

There are clear-cut differences in the stoichiometry parameter (n) between 3-MP and compound 2 binding. The n values for 3-MP are around 2 and for compound 2 are close to 1. This indicates that there exist only one binding site for compound 2 in both isoenzymes but two binding sites for 3-MP, also in both isoenzymes.

## Discussion

In a previous work [9] we found two isoenzymes in porcine PEPCK-C that differed considerably in their kinetic and functional properties. Those differences were translated into considerable differences in pig and pork phenotypic traits of considerable economic relevance. In this work we have continued the biochemical characterization of both isoenzymes and we have used them as a model system to search for new inhibitors able to modulate PEPCK-C activity due to the relevance of PEPCK-C activity in pathological processes.

To further investigate the catalytic differences between PEPCK-C isoenzymes 139Met and 139Leu we decided to use a known inhibitor, 3-MP, one of the first specific inhibitors of PEPCK-C described [15]. In our previous work [9], the largest kinetic differences between pig isoenzymes were detected in their interaction with nucleotides; here again both isoenzymes show the largest differences when the inhibition is studied varying a nucleotide, GTP. This means that the Met139Leu substitution affects probably the structure of the enzyme active site around the nucleotide binding site.

We have found the rather uncommon uncompetitive inhibition pattern of both PEPCK-C isoenzymes when both nucleotides (GDP and GTP) were used as variable substrate. This inhibition pattern requires a prior binding of the substrate to the active site in order to make the inhibitor site accessible. Recently, Balan et al. [23] found a very similar result studying the inhibition of rat PEPCK-C by 3-MP using both kinetic experiments and the determination of the 3D structure of the protein with bound 3-MP. They find uncompetitive inhibition when using GDP or GTP as variable substrates. However, their structural determinations showed that 3-MP had two binding sites: one in the active site that was responsible for the competitive component of their kinetic experiments, and the other one close to the active site and able to influence allosterically the binding of substrates and inhibitors to the active site. These structural data are not compatible with the apparent uncompetitive character of 3-MP inhibition because uncompetitive inhibition requires the existence of independent (or different) substrate and inhibitor binding sites [24]. Balan et al. [23] explain their results suggesting that the anomalous uncompetitive inhibition detected by kinetic means is due to the communication between the allosteric inhibitor site and the active site. Whatever is the true nature of the inhibition by 3-MP, this inhibitor serves to demonstrate the existence of further biochemical differences between pig PEPCK-C isoenzymes because the comparison of K_i_ values for 3-MP in the presence of GTP showed strong differences between pig isoenzymes. 139Met has an almost three fold larger affinity for 3-MP than 139Leu (273 vs 873 μM). We found no difference between PEPCK-C isoenzymes when analyzing 3-MP effects on PEP binding and only slight ones when analyzing its effects on OAA binding (K_i_ for139Leu is 50% larger than for 139Met). The inhibition mode for PEP and OAA was the same for both isoenzymes and substrates, a mixed non-competitive one.

The screening of the chemical library with 10,000 compounds yielded two positive compounds that stabilize the enzyme increasing the T_m_ around 8°C. The positive compounds were N1-cyclohexyl-2-{1-[4-methyl-2-(2-thienyl)-1,3-thiazol-5-yl]ethylidene}hydrazine-1-carbothiamide, or compound 1, and N'1-({5-[1-methyl-5-(trifluoromethyl)-1H-pyrazol-3-yl]-2-thienyl}methylidene)-2,4-dichlorobenzene-1-carbohydrazide, or compound 2.

In general, the effects of both compound 1 and 2 on *V*_max_ are stronger than on *K*_m_ values, which suggests that their inhibition mode is different from that of 3-MP and that they could have a different binding site than 3-MP. On the other hand, the structural similarity of compounds 1 and 2 (Fig 4) suggest these two compounds may share a common binding site.

There were quantitative differences between the inhibitory effects of compound 2 on 139Met and 139Leu: using OAA as a variable substrate, *V*_max_ values were 4.6 and 12.7 μmol min^-1^ mg^-1^ for 139Met and 139Leu, respectively. This significant difference showed that 139Met was more sensitive to compound 2 than 139Leu. Another significant difference when using GTP as a variable substrate was that *K*_m_ value of 139Met raised 2.5-fold in presence of compound 2, whereas 139Leu *K*_m_ decreased around 30%. As we report with 3-MP, compound 2 constitutes a valuable tool to investigate the biochemical differences between pig isoenzymes.

The results found in the ITC experiments show again differences between PEPCK-C isoenzymes when analyzing GTP binding. As it happened in the kinetic experiments (see Table 1), K_d_ for 3-MP of 139Met was much lower than that of 139Leu (19 vs 39 μM) in the presence of GTP, which resembles the effects found on K_i_ values in the kinetic experiments. ITC experiments showed also very different stoichiometry when studying 3-MP and compound 2 binding. Whereas n was 1 in all experiments using compound 2, we found a value of 2 when using 3-MP. As this parameter reflects the number of binding sites, our results suggest that there is only one binding site for compound 2 but two sites for 3-MP, in agreement with two 3-MP binding sites reported in the crystallographic structure of rat PEPCK-C by Balan et al. [23].

The study by ITC of compound 2 binding resembles also the kinetic results (see Table 3): the affinity of the 139Met isoenzyme (19 μM) for compound 2 in the presence of GTP is slightly lower than that of the 139Leu isoenzyme (14 μM).

The existence of notable differences between K_d_ values for both isoenzymes in the presence and in the absence of GTP (Table 4) confirms that both inhibitors affect the nucleotide binding site. The sites of 3-MP binding have been already determined by Balan et al. [23]; our results with compound 2 do not allow us to specify where the binding site for this inhibitor is located.

Most PEPCK-C effectors described in the literature are substrate analogues acting mainly as competitive inhibitors [15]. Finding new PEPCK-C inhibitors is important because of the PEPCK-C role in diabetes and cancer. PEPCK-C has been identified as a major contributing factor for the appearance of diabetes mellitus type 2 [25]. Cancer cells may overexpress PEPCK-C to avoid the decreased levels of glucose due to the preponderance of glycolysis in tumor areas [6]. New effectors with different patterns of inhibition could be interesting due to the potential existence of synergistic effects between inhibitors with different binding sites.

IC_50_ values of compounds 1 and 2 are rather low which means that these inhibitors could be used as *in vivo* effectors. However, their poor solubility and their slight interaction with auxiliary enzymes indicate that compounds 1 and 2 are not very selective inhibitors and that they would need some improvement before they can be used as *in vivo* PEPCK-C effectors. Directed chemical modification of compounds 1 or 2 to improve their solubility and specificity could yield suitable effectors against diabetes or cancer.

In conclusion, the use of inhibitors reveals further biochemical differences between pig PEPCK-C isoenzymes reinforcing the idea that the Met139Leu substitution affects mainly the GTP/GDP binding site. The new PEPCK-C inhibitors found in this study could be helpful to modulate PEPCK-C activity *in vivo* if their solubility and selectivity could be improved.

## Supporting Information

S1 Fig Concentration-dependent inhibition of PEPCK-C by 3-MP. Data are the mean of three independent experiments ± SD.
(TIF)

S2 Fig Dixon plots of 3-MP adding PEP and GDP as variable substrates. (A) Dixon plot of 139Met varying PEP. (B) Dixon plot of 139Leu varying PEP. (C) Dixon plot of 139Met at saturating concentration of GDP. (D) Dixon plot of 139Leu at saturating concentration of GDP.
(TIF)

S3 Fig Dixon plots of 3-MP adding OAA and GTP as variable substrates. (A) Dixon plot of 139Met varying OAA. (B) Dixon plot of 139Leu varying OAA. (C) Dixon plot of 139Met at saturating concentration of GTP. (D) Dixon plot of 139Leu at saturating concentration of GTP.
(TIF)

S4 Fig Concentration-dependent inhibition of PEPCK-C by compounds 1 and 2. Data are the mean of three independent experiments ± SD. Compound 1 plot (A) was adjusted to linear regression due to its precipitation above 200 μM.
(TIF)
