# Supplementary figures and images for: Inhibition of Pig Phosphoenolpyruvate Carboxykinase Isoenzymes by 3-Mercaptopicolinic Acid and Novel Inhibitors
Source: PLoS One. 2016 Jul 8;11(7):e0159002. doi: 10.1371/journal.pone.0159002 (PMC4938538; doi:10.1371/journal.pone.0159002)

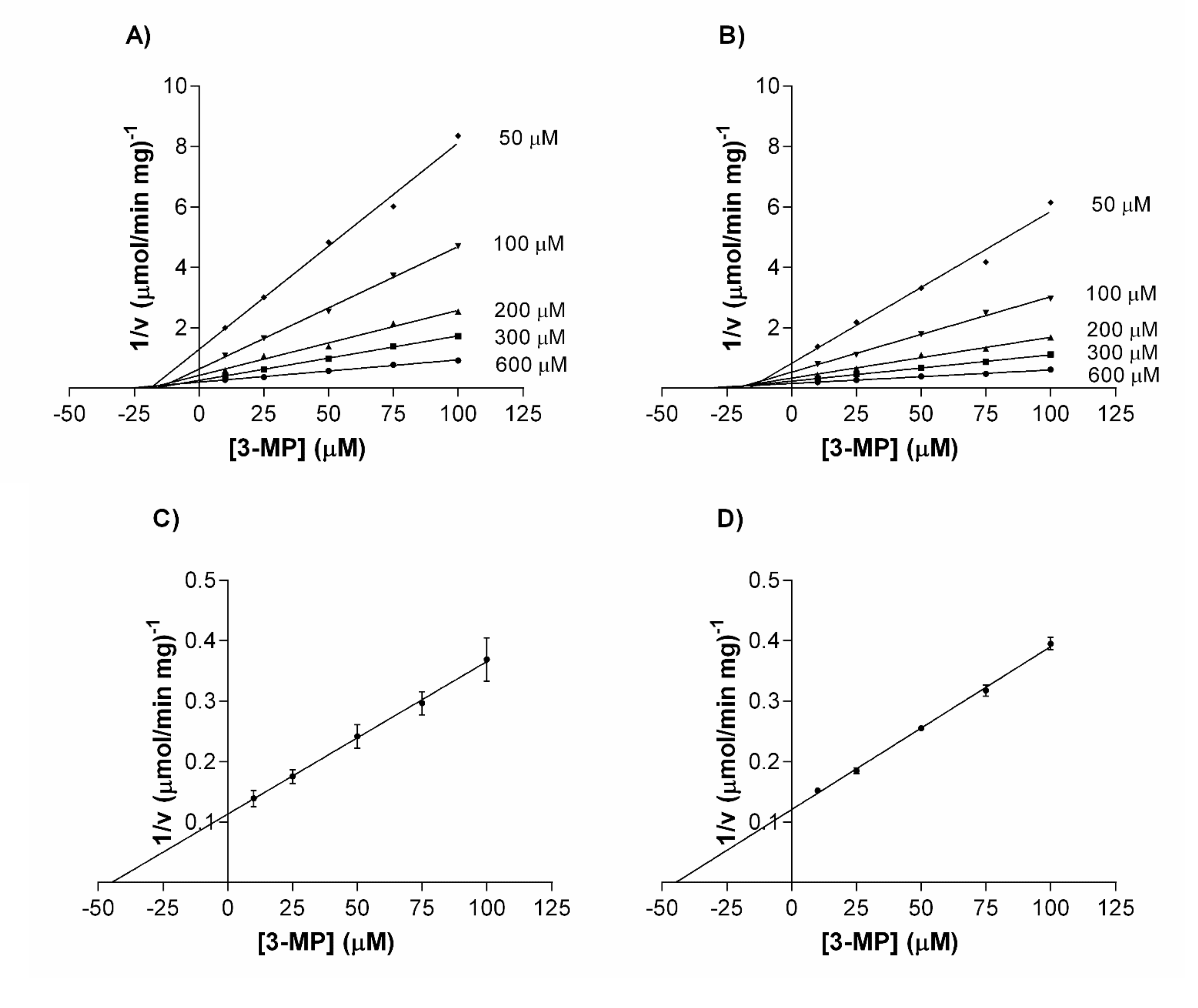

Supplement: S2 Fig — (A) Dixon plot of 139Met varying PEP. (B) Dixon plot of 139Leu varying PEP. (C) Dixon plot of 139Met at saturating concentration of GDP. (D) Dixon plot of 139Leu at saturating concentration of GDP. (TIF) [file pone.0159002.s002.tif]

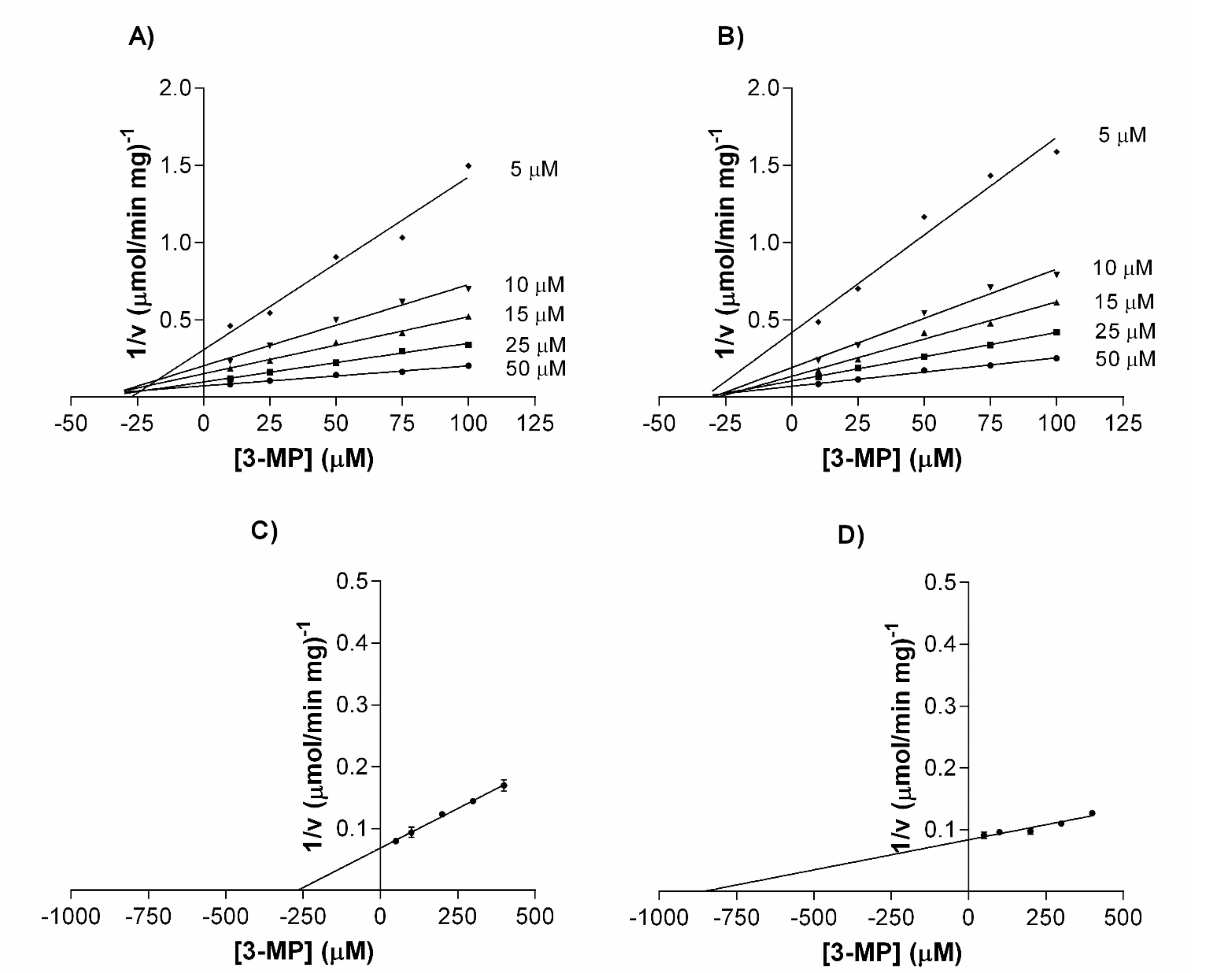

Supplement: S3 Fig — (A) Dixon plot of 139Met varying OAA. (B) Dixon plot of 139Leu varying OAA. (C) Dixon plot of 139Met at saturating concentration of GTP. (D) Dixon plot of 139Leu at saturating concentration of GTP. (TIF) [file pone.0159002.s003.tif]

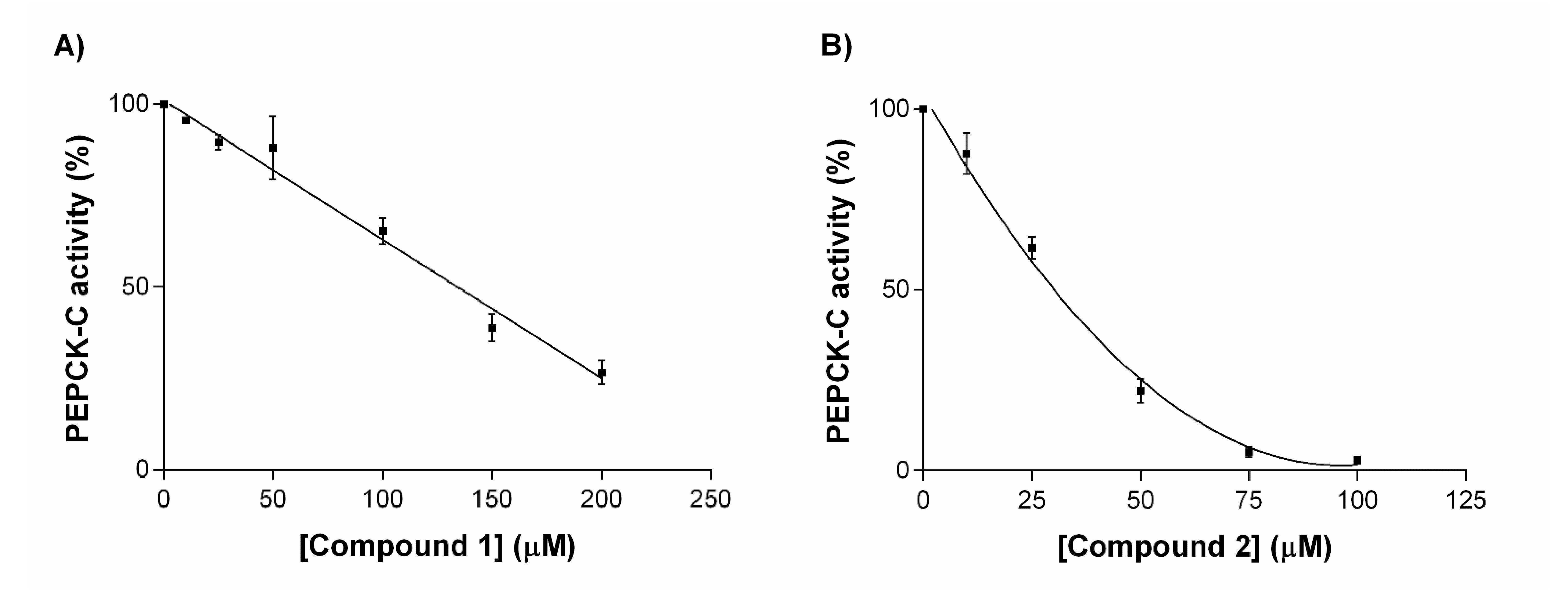

Supplement: S4 Fig — Data are the mean of three independent experiments ± SD. Compound 1 plot (A) was adjusted to linear regression due to its precipitation above 200 μM. (TIF) [file pone.0159002.s004.tif]
